# A Liquid Mirror Resonator

**DOI:** 10.3390/mi14030624

**Published:** 2023-03-08

**Authors:** Elad Haber, Mark Douvidzon, Shai Maayani, Tal Carmon

**Affiliations:** 1 Mechanical Engineering, Technion-Israel Institute of Technology, Haifa 3200003, Israel; eladhaber@gmail.com; 2 Mark Douvidzon, Solid State Institute and Physics Department, Technion-Israel Institute of Technology, Haifa 3200003, Israel; 3 Research Laboratory of Electronics (RLE), Massachusetts Institute of Technology, Cambridge, MA 02139, USA; 4 School of Electrical Engineering, Tel Aviv University, Tel Aviv 6997801, Israel

**Keywords:** surface science, capillary waves, cavity optocapillaries, thermal capillary waves

## Abstract

We present the first experimental demonstration of a Fabry‒Perot resonator that utilizes total internal reflection from a liquid–gas interface. Our hybrid resonator hosts both optical and capillary waves that mutually interact. Except for the almost perfect reflection by the oil–air interface at incident angles smaller than the critical angle, reflections from the liquid-phase boundary permit optically examining thermal fluctuations and capillary waves at the oil surface. Characterizing our optocapillary Fabry‒Perot reveals optical modes with transverse cross-sectional areas of various shapes and longitudinal modes that are separated by the free spectral range. The optical finesse of our hybrid optocapillary resonator is Fo = 60, the optical quality factor is Qo = 20 million, and the capillary quality factor is Qc = 6. By adjusting the wavelength of our laser near the optical resonance wavelength, we measure the liquid’s Brownian fluctuations. As expected, the low-viscosity liquid exhibits a distinct frequency of capillary oscillation, indicating operation in the underdamped regime. Conversely, going to the overdamped regime reveals no such distinct capillary frequency. Our optocapillary resonator might impact fundamental studies and applications in surface science by enabling optical interrogation, excitation, and cooling of capillary waves residing in a plane. Moreover, our optocapillary Fabry‒Perot might permit photographing thermal capillary oscillation, which the current state-of-the-art techniques do not support.

## 1. Introduction

Interfacial tension and capillary waves are relevant to a variety of microfluidic systems, among them compound droplets [1], liquid interfaces [2], emulsification mechanisms [3], droplets coalescence [4,5], microfluidic devices [6], bioreactors [7], bifluid flows [8], microjets [9], and particle separation [10]. In a seemingly unrelated lane of study, optical microfluidics [11,12] have been used for biological and chemical analysis [13,14,15,16] as well as for biolasers [17]. Furthermore, the synergy between photonics and microflucids permitted the optical excitation of sound [18], vibration [19,20], and flows [21] in optical resonators, as well as the mapping of optical modes of droplets [22]. While sound and light are common to all states of matter, capillary waves are unique to the liquid phase of matter. Recently, droplet resonators [23] were reported where light and capillary waves interact [24,25]. Here, we extend such droplet optocapillary cavities to a standard Fabry‒Perot resonator, giving optical access to flat liquid‒gas interfaces and to the capillary waves residing there.

**Figure 1:**
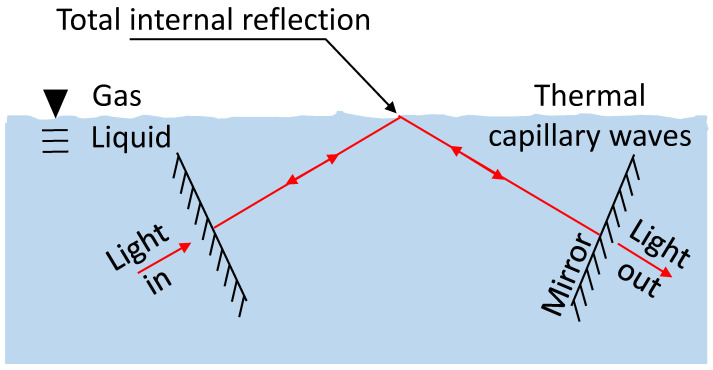
**Schematic description** of our resonator, where we use total internal reflection from a gas‒liquid interface as one of the reflectors. As such, the cavity roundtrip length is affected by thermal capillary waves at the liquid-phase boundary.

In detail, as shown in Figure 1, surface energy produces tension at the liquid‒gas interface. As such, an oil–air surface behaves like a spring or a membrane, attempting to bring the surface back to mechanical equilibrium. In a vessel containing a liquid, the mechanical equilibrium of the liquid-phase boundary is a flat surface. On the other hand, random thermal fluctuations perturb the gas‒liquid interface to deviate it from its flat state. Overall, our liquid‒gas interface fluctuates in a Brownian manner while totally internally reflecting light and serving as one of the mirrors of our resonator. In the following sections, we will discuss the capacity of an optical resonator to measure variation in optical path length, followed by a brief overview of capillary waves, specifically Brownianly excited waves.

### 1.1. Optics: A Resonator with a Moving Mirror

Interferometric techniques are typically limited in achieving precision in size measurement. Generally, the resolution limit of interferometric techniques is comparable to the optical wavelength and provides micron-scale accuracy. Thermal capillary waves have an amplitude that is too small to examine using interferometric techniques. Using optical resonators in place of interferometers can significantly improve the resolution of size measurements toward the nanometer range. In principle, optical resonators can measure changes in optical path length, with a wavelength resolution divided by the optical quality factor [24].

### 1.2. Fluid Mechanics and Surface Science: Capillary Waves

Capillary waves are perturbations in liquid level, or height, that propagate along the interface between two fluids, typically gas and liquid. For example, capillary waves of various amplitudes and wavelengths can propagate at the interface between oil and air. The restoring force applied by surface tension or gravity on a liquid wave pushes the liquid level towards a mechanical equilibrium. For long waves, the restoring force is gravity, while for short waves, the restoring force is surface tension. The unique behavior of liquid waves relates to their dispersive character. Unlike sound waves or light waves in a nondispersive medium, the velocity of a capillary wave depends on its wavelength. The dispersion relation for a capillary wave is given by [26]:

(1) $${Ω}^{2}=gk+\gamma \frac{k^{3}}{\rho }$$

where Ω is the angular frequency, *g* is the acceleration of gravity, *k* is the wavenumber, *γ* is the surface tension, and *ρ* is the liquid mass density. Using v=Ωk , we obtain that the phase velocity for the capillary wave is

(2) $$v_{phase}=\sqrt{\frac{g}{k}+\frac{\gamma k}{\rho }}$$

**Figure 2:**
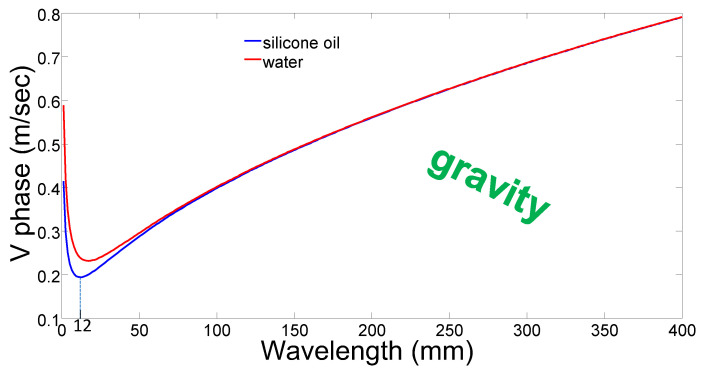
**Speed as a function of wavelength** for capillary waves at the liquid–air interface, for two different liquids: silicone oil (blue) and water (red).

As shown in Figure 2, for gravity waves, longer waves propagate faster than short waves. Conversely, for short waves where surface tension functions as the major restoring force, shorter waves propagate faster. Accordingly, there is a minimum speed at which the wave can go. For silicone oil, which we use here, the minimum speed is approximately 0.2 m/s, which occurs for a 12 mm wavelength. Similarly, fluctuations at rates higher than 17 Hz in the frequency domain relate, here, to the parameter space where surface tension is the dominant restoring force, while rates lower than 17 Hz relate to gravitational restoring forces. The group velocity, which is the velocity at which the envelope of the waves propagates, is defined as vgroup=dωdk. Using Equation (1) leads to a group velocity.

(3) $$v_{group}=\frac{g+3\gamma k^{2}/\rho }{2\sqrt{\left(gk+\gamma k^{3}/\rho \right)}}$$

For gravity waves, the group velocity is half of the phase velocity, but for waves where surface tension dominates, the group velocity is approximately 1.5 higher than the phase velocity.

### 1.3. Statistical Mechanics: Thermal Capillary Waves

Thermal capillary waves represent a phenomenon related to the effects of Brownian motion of the liquid-phase boundary. For instance, the water‒air interface in a cup of water tends to be viewed as a smooth stationary surface, but will actually look like a stormy sea if monitored with an angstrom resolution. The estimation of the amplitude related to the thermal capillary wave can be performed by using the equipartition theorem [27],

(4) $$\frac{1}{2}k_{B}T=\frac{1}{2}K\Delta x^{2}$$

where $k_{B}$ is the Boltzmann constant, T is the absolute temperature, K is the spring constant, and Δx is the displacement from mechanical equilibrium. Expressing the spring constant, K, using surface tension γ and considering surface tension on the order of 10 to 100 mN/m for most liquids leads to an interface roughness on the order of

(5) $$\langle \Delta x\rangle =\sqrt{k_{B}T/\gamma }=3\sim 5Å$$

at room temperature. Regular optics cannot resolve this scale, since it resides below the diffraction limit of imaging systems. Additionally, photographing techniques that use optical interference fail to image such thermal capillary waves. Thermal capillary waves were theoretically predicted [28] and were seen in indirect ways, such as through light [29] and X-ray scattering [30,31,32,33], as well as in systems simulating liquids [27]. Recently, droplet micro-optocapillary resonators have allowed the optical interrogation [1] of capillary waves and even the optical excitation [25] of capillary resonances. One can call such stimulated excitation of capillary waves a water-wave laser [25], analogous to Brillouin lasers [34], where stimulated emission is mediated by sound.

Summarizing this section, optocapillary resonators can mutually host optical and capillary waves, while their interaction is resonantly enhanced. Our Fabry‒Perot resonator permits the optical interrogation of common flat liquid surfaces, including those in liquid-containing vessels. Furthermore, our optical interrogation of the flat liquid level benefits from resonance enhancement with an optical quality factor exceeding 20 million. This work extends the capacity of microfluidic devices to access capillary waves and detect thermal fluctuations in the total internal reflection region. We believe that our device will soon permit pump and probe experiments, where one light beam excites or cools capillary waves while the other measures their amplitude.

## 2. Experimental Setup

**Figure 3:**
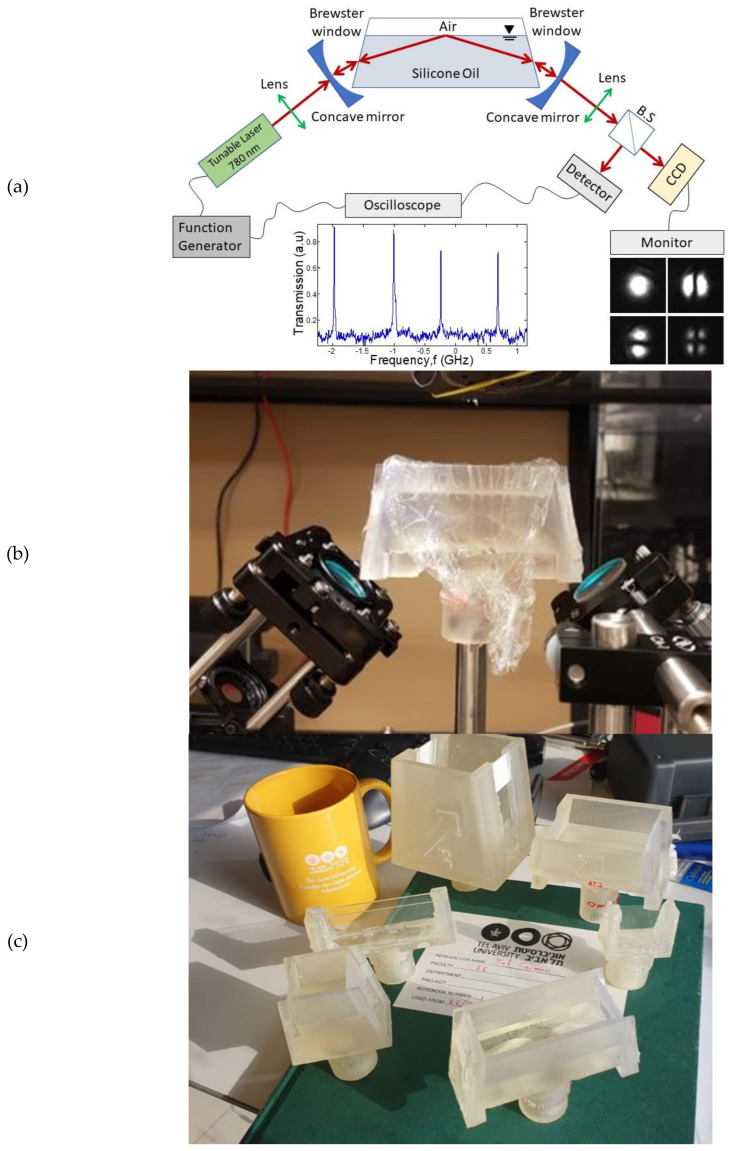
**Experimental setup**. (**a**) Detailed description; (**b**) the liquid container and the concave mirrors. (**c**) Several models of the liquid containers.

Our optocapillary cavity (Figure 3) is based on a Fabry‒Perot resonator, where one of the reflectors relies on the total internal reflection from a liquid-phase boundary. The other two reflectors are high-reflectivity dielectric concave mirrors, with a reflectivity of 99.97%. The radius of curvature for these mirrors fulfils the stability condition for such a resonator [35]. A designated container to hold the liquid was printed using a 3D printer, and two microscope slides were affixed to serve as Brewster windows. Brewster windows’ advantage is their 100% transmission, achieved with no need for anti-reflection coating. A 770 to 790 nm tunable laser is free-space coupled into the resonator through one of the concave mirrors. The light polarization is controlled by a half-wave plate on a rotary mount. Polarization is adjusted so that minimal reflection bounces back from the Brewster windows. The other concave mirror functions as the output coupler. A beam splitter sends the transmitted light to both an amplified photodetector and a camera. In this manner, we can simultaneously monitor the spectral and spatial properties of the optical mode.

To excite an optical mode, the laser’s optical frequency is scanned through the optical resonances of the Fabry‒Perot. Resonance conditions are achieved when the optical roundtrip length equals an integer number of wavelengths of light. Reaching this condition allows light to be transmitted out of the cavity and photographed by the camera.

Scanning the laser gives rise to optical resonances spaced almost evenly in frequency in every free spectral range, with some deviation originating from thermal capillary waves. When operating the laser at a wavelength for which some of the light can be transmitted, a slight change in the liquid level (due to Brownian motion) changes the transmission of the optical resonator by drifting the resonance wavelength relative to the laser wavelength.

Two main properties should be considered when choosing a liquid for the experiment. From an optics point of view, the liquid should be as transparent as possible to achieve high optical finesse and high optical quality. Therefore, we chose silicone oil, which allowed optical quality factors higher than one billion [36].

From a fluid mechanics point of view, and as evident from Equation (5), the surface tension must be as low as possible to obtain high-amplitude thermal capillary waves. Silicone oil, in this regard, has a surface tension of γ= 20.8 mNm, which is approximately half of the water’s surface tension. Another property that influences capillary wave damping is the viscosity of the liquid. Three different viscosities were used in this experiment, 50, 350, and 60,000 cSt, which allowed the examining of our system in the underdamped, critically damped, and overdamped regimes.

## 3. Experimental Results

### 3.1. Characterization

**Figure 4:**
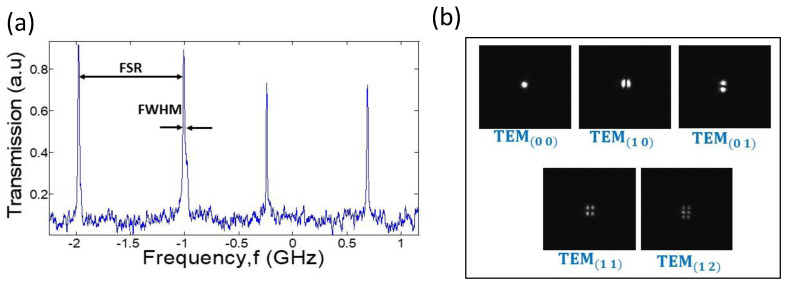
**Resonator characterization.** (**a**) Spectral distribution of the optical mode. (**b**) Spatial mode profile along the direction transverse to the light propagation direction. The viscosity is 60,000 cSt.

We scanned the laser through several cavity resonances. This scan is fast to prevent the effects of capillary waves. As expected, at wavelengths that fulfil the resonance conditions, the transmission through the resonator increased, as detected by a camera and a photodiode. Figure 4 shows some of the modes that were detected by a photodiode. The free spectral range is 850 ± 13 MHz, where the error stands for the standard deviation. Figure 4 also shows photographs of the various transverse modes detected by a camera. Here, the transverse modes have a rectangular symmetry of the Hermite–Gaussian form. These kinds of modes are expected in a resonator with Brewster windows. Analyzing the results in Figure 4 reveals an optical finesse of 60 and an optical quality factor, Qo, of 2 × 10^7^. The optical roundtrip length in our resonator is 300 mm.

Strong transmission peaks (Figure 4, left) appear simultaneously with a TEM(00) mode, as photographed by the camera. Higher-order transverse modes, such as TEM(11), were photographed by the camera (Figure 4, right) but are hardly seen in the spectrum because of their relatively low overlap with the laser mode shape.

Unlike solid-based resonators, where the free spectral range is typically not changing in time, we see in Figure 4 a nonregular separation between the modes. To give a scale, the 13 MHz standard deviation in the optical resonance frequency corresponds to a 10.1 nm extension in the optical roundtrip length of the cavity. These types of deviation will be parametrically studied in what follows, while changing the optical scan speed and liquid viscosity.

### 3.2. Spectral Response for Various Viscosities

We now study our resonator by analyzing its spectral response in a set of three experiments where we change the viscosity. To keep all liquid properties unchanged, except for viscosity, we use silicone oil in all of these experiments. The length of the silicone molecule determines liquid viscosity while keeping the optical transparency, specific weight, and surface tension almost the same during the different experiments.

In detail, we repeat the same experiment with three different viscosities.

- Low viscosity (50 cSt), corresponding to underdamped capillary oscillation (capillary quality factor, Qc > 1);
- Medium viscosity (350 cSt), corresponding to critically damped capillary oscillations (Qc ≈ 1);
- High viscosity (60,000 cSt), corresponding to overdamped capillary oscillations (Qc << 1).

**Figure 5:**
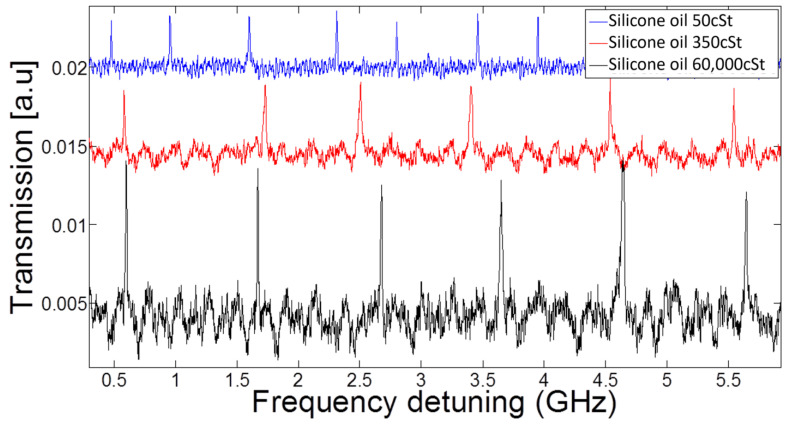
**Viscosity comparison.** Transmission vs. frequency detuning for three different viscosities of silicone oil. Laser scans at 12 GHz per second. The fluid density is ρ=960 kg/m^3,^ and the surface tension is γ=20.8 mNm. The scan duration is 0.5 s.

For the highest viscosity (Figure 5), the free spectral range is 986 ± 78 MHz, where error stands for standard deviation. This is close to the free spectral range of 996 ± 160 MHz, measured for the medium viscosity.

However, going to low viscosity, where the system is in the underdamped region, our capillary resonator was expected to oscillate sinusoidally in time. For this reason, the resonance frequency sweeps back and forth and, therefore, has additional passages through resonance conditions during the scan, as indicated by the extra peaks in the blue plot. This issue will be investigated in more detail in the following section.

### 3.3. Measuring Thermal Fluctuations for Various Viscosities

**Figure 6:**
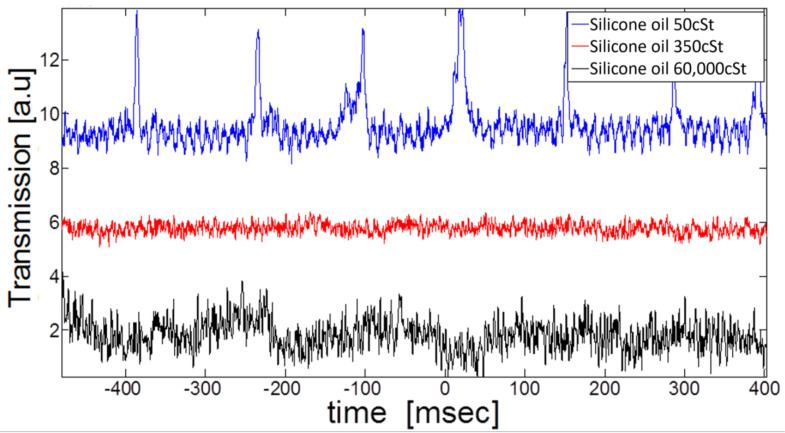
**Resonator characterization.** (left) Modes as detected by a photodiode. (right) Higher-order transverse modes as detected by a camera.

To isolate the effect of the thermal sinusoidal capillary oscillations, we stop the laser wavelength scan so that thermal fluctuations are the single nonstationary element in our experiment. As expected, and because of thermal capillary oscillations, the low viscosity exhibits the passage of the cavity resonances via the laser wavelength every 128 ± 18 ms (Figure 6), corresponding to a frequency of 7.97 ± 1.23 Hz. Since the optical resonance sweeps twice (going back and forth) through the laser wavelength during one capillary cycle, the dominant thermal capillary oscillation frequency is 3.98 ± 0.61 Hz. Estimating the capillary quality factor, Qc, from the deviation in resonance frequency reveals Qc = 3.98/0.61 = 6.44, which indicates operation in the underdamped regime. As expected, going to higher viscosities reveals no distinct frequency of capillary resonances.

In its straightforward realization, the first capillary modes are similar to a guitar string, whose length equals half the wavelength. Along these lines, and using Equation (2), the measured capillary frequency (3.98 Hz) corresponds to a capillary resonator length equal to 49.7 mm. In the experiment, our capillary resonances reside in a 63 × 25 mm rectangle, close to within a form factor of our calculated length (49.7 mm).

## 4. Conclusions

Here, we build the first resonator with one of its mirrors based on total internal reflection for a liquid‒gas interface. At low viscosities, the thermal fluctuation of the liquid-phase reflector generates spontaneous drifts in the optical path length accompanied by the casual excitation of optical resonances, despite the stationary optical input. Our optocapillary resonator opens future research directions, including optical excitation, interrogation, and cooling of capillary waves.

## Data Availability

Data is avaliable by contacting the first author.
